# Coagulation dysfunction events associated with novel tetracycline-class drugs: a disproportionality and Bayesian analysis based on the FAERS database

**DOI:** 10.3389/frabi.2026.1781902

**Published:** 2026-06-05

**Authors:** Hongxin Li, Jun Wang, Lingjun Kong, Qingwang Wei, Chengwu Shen, Dongna Zou

**Affiliations:** 1 Department of Pharmacy, Liaocheng People′s Hospital, Liaocheng, Shandong, China; 2 Department of Pharmacy, Shandong Provincial Hospital Affiliated to Shandong First Medical University, Jinan, China

**Keywords:** coagulation dysfunction, data mining, FDA adverse event reporting system (FAERS), novel tetracycline-class drugs, reporting odds ratio

## Abstract

**Introduction:**

In contemporary clinical practice, novel tetracycline-class antimicrobial agents have been increasingly utilized due to their favorable pharmacological profiles. However, the potential association between novel tetracycline-class drugs and coagulation dysfunction is relatively underreported in the literature. This study utilizes the FDA Adverse Event Reporting System (FAERS) to identify and analyze risk signals for coagulation disorders associated with three commonly used the novel tetracyclines in clinical practice. The findings are expected to provide valuable references for clinicians in the targeted monitoring of adverse drug reactions.

**Methods:**

Coagulation dysfunction reports associated with the novel tetracycline-class drugs, submitted to the FDA Adverse Event Reporting System (FAERS) from January 2004 to December 2024, were collected. Data mining was performed using the ROR, PRR, BCPNN, and MGPS. All data extraction was conducted using R software (version 4.4.2).It should be noted that FAERS is a spontaneous reporting system without exposure denominators and is subject to reporting bias and confounding factors.

**Results:**

A total of 346 coagulation dysfunction reports with the novel tetracycline-class drugs as suspected drugs were screened, including 327 for tigecycline, 6 for omadacycline, and 13 for eravacycline. Disproportionate reporting signals of coagulation dysfunction were detected for both tigecycline and eravacycline, with tigecycline showing the strongest signal [ROR = 46.85 (42.29-51.91)]. Subgroup analyses by age, gender, and dosage revealed a higher proportion of male patients receiving tigecycline and eravacycline. while tigecycline and eravacycline exhibited a stronger disproportionate reporting signal in male patients over 65 years old. Omadacycline showed stronger disproportionate reporting signal at a daily dose of 100 mg. The peak onset time for omadacycline was consistently 9 days. However, all subgroup findings for omadacycline and eravacycline should be interpreted with extreme caution due to the limited number of reports. These observations are considered exploratory rather than confirmatory.

**Conclusion:**

Disproportionality analysis identified prominent signals of coagulation dysfunction signals associated with tigecycline and eravacycline. Distinctive reporting patterns of coagulation abnormalities were observed across the three novel tetracyclines, with visible discrepancies in patient age, gender, therapeutic indications, daily dosage, clinical outcomes, and adverse event onset time. Based on the above findings, clinicians should maintain high vigilance against the potential coagulation abnormalities induced by novel tetracyclines, and implement standardized and rational therapeutic drug monitoring during clinical medication practice.

## 1 Introduction

In recent years, multidrug-resistant Gram-negative bacilli (MDR-GNB) infections have emerged as a major global health challenge (Scudeller et al., 2021). The increasing prevalence of MDR-GNB, including carbapenem-resistant Enterobacterales, Pseudomonas aeruginosa, and Acinetobacter baumannii, has significantly limited the therapeutic of conventional antimicrobial agents, leading to prolonged hospital stays, increased mortality rates, and rising medical costs. Against this backdrop, the development of novel antimicrobial agents with potent activity against MDR-GNB has become an urgent clinical need. These structural modifications not only enhance their antimicrobial spectrum and potency against MDR-GNB but also reduce the risk of drug resistance associated with traditional tetracyclines, overcoming the limitations of earlier tetracycline agents that were rendered ineffective by efflux pumps and ribosomal protection mechanisms. Through structural modification of the side chains of tetracycline antibiotics, novel tetracycline-class agents including tigecycline (glycylcycline), omadacycline (aminomethylcycline), and eravacycline (fluorocycline) have been developed. These agents serve an important role in the management of infections caused by associated with multidrug-resistant bacteria (Kim and McElvania, 2023). Novel tetracycline-class agents demonstrate distinctive clinical efficacy in managing infections associated with multidrug-resistant and pan-drug-resistant bacteria, including complicated intra-abdominal infections, hospital-acquired pneumonia, ventilator-associated pneumonia, and bloodstream infections. Owing to their favorable pharmacokinetic and pharmacodynamic properties, as well as their proven clinical efficacy, these agents are widely used in clinical practice and have been recommended by multiple international and domestic expert consensus and clinical practice guidelines (Guan et al., 2016; Tamma et al., 2021), serving as a critical therapeutic option for patients with limited antimicrobial choices.

With the widespread clinical application of novel tetracycline-class drugs, the safety profile of these therapeutics have attracted increasing attention from clinicians and researchers. Notably, reports of drug-associated coagulopathy have gradually increased in recent years (Sabanis et al., 2015; Guo et al., 2022; Qian et al., 2023; Zhang et al., 2023; Xv and Tan, 2024), including hypofibrinogenemia, prolonged activated partial prothrombin time (APTT), prolonged prothrombin time, and even life-threatening bleeding events in severe cases. These adverse reactions have raised concerns about the safe use of novel tetracycline-class drugs, particularly in patients with underlying coagulation disorders or those receiving concurrent anticoagulant therapy.

Importantly, to date, no FAERS-based studies have been conducted on the novel tetracycline-class drugs in the coagulation system, highlighting the novelty and clinical significance of the present study focused on exploring the association between novel tetracyclines and coagulation abnormalities using FAERS data. In this study, a comprehensive analysis of positive AEs associated with the coagulation system was conducted by mining the FAERS database. Additionally, the correlations between therapeutic agents and AEs were visualized using graphical representations, aiming to provide a reference for clinical medication and offering preliminary evidence for subsequent targeted research. The time to onset of coagulopathy was further analyzed in the general population and within each age, gender, indication, daily dose, time to onset, and outcome indicators, which has not been previously reported. The results of this study may assist clinicians and healthcare policymakers in the targeted surveillance of adverse drug reactions and inform evidence-based recommendations for the safe and rational clinical application of novel tetracycline-class agents.

## 2 Materials and methods

Data Collection: A retrospective study was conducted using R software on relevant cases in FAERS from January 2004 to December 2024, Given the lack of uniform standards for drug naming within the FAERS system, coupled with the diverse backgrounds of reporters and the variety of report formats, multiple names for the same drug can exist within the system. Therefore, this study utilized the NCBI(National Center for Biotechnology Information) database (https://www.ncbi.nlm.nih.gov/) to search and compile the generic names, brand names, and research codes of these tetracyclines approved by the FDA as of December 2024. By employing the compiled list (**Supplementary Table 1**) to conduct searches in the FAERS database, the objective was to extract relevant drug data as comprehensively as possible, thereby ensuring data integrity. The Medical Dictionary for Regulatory Activities (MedDRA) constitutes a standardized international medical terminology, which facilitates the global documentation and reporting of adverse drug events (ADEs). MedDRA adopts a hierarchical classification framework covering multiple levels from granular low-level terms to the System Organ Class (SOC). As the highest hierarchical level in MedDRA, the SOC is widely applied to the classification and reporting of adverse events within pharmacovigilance monitoring and reporting systems. To systematically summarize and analyze ADE profiles of coagulopathy, all included coagulopathy-related ADEs were coded with MedDRA Preferred Terms (PTs) and subsequently mapped to the corresponding SOC level using MedDRA Version 27.1. All retrieved PTs related to coagulation and hemostasis disorders were included to form the composite endpoint of coagulation dysfunction (Guo et al., 2022). The primary outcome was defined as overall coagulation-related adverse events. The secondary outcomes included frequently reported coagulation dysfunction events, specifically: hypofibrinogenemia, prolonged activated partial thromboplastin time (APTT), prolonged thrombin time (TT), prolonged prothrombin time, abnormal prothrombin time, prolonged clotting time, coagulation disorders, decreased fibrinogen, blood fibrinogen, blood fibrinogen abnormal, activated partial thromboplastin time abnormal, activated partial thromboplastin time prolonged, coagulation time, coagulation time abnormal, prothrombin time, thrombin time, thrombin time abnormal, and thrombocytopenia (Brown, 2003). The primary outcome was defined as coagulation factor-related dysfunction dominated by hypofibrinogenemia. Thrombocytopenia was included as a secondary hemostasis-related outcome. Although thrombocytopenia represents a platelet hematologic abnormality rather than direct coagulation factor impairment, it was incorporated to comprehensively assess the overall bleeding and hemostatic risk associated with novel tetracyclines, given its close clinical correlation with coagulation disorders and bleeding events. All data extraction was conducted using R software (version 4.4.2). R is a statistical programming language that enables retrieval of adverse drug reaction (ADR) and drug-related information via customized algorithms.

Data Processing: This retrospective pharmacovigilance analysis was conducted using the FAERS database. FAERS is a compilation of adverse drug event (ADE) reports and allows researchers to perform signal detection and quantify the associations between drug dosing and ADEs. The FAERS database is updated quarterly and comprises seven datasets on demographic and administrative information (DEMO), drug information (DRUG), adverse drug reaction information (REAC), patient outcomes information (OUCT), reported sources (RPSR), drug therapy starts dates and end dates (THER), and indications for drug administration (INDI). There are unavoidable cases of duplicate reporting in FAERS due to the characteristics of data updating. Deduplication followed FDA-recommended methods, using the DEMO table (key fields: PRIMARYID, CASEID, FDA_DT) and DRUG table (storing ‘Role_Code’) from FAERS QDFs [Version 24Q4], linked via CASEID. Reports were selected from the DEMO table using the three fields, sorted descendingly by CASEID, FDA_DT, and PRIMARYID. Duplicates were resolved by retaining the report with the largest FDA_DT (for same CASEID) or PRIMARYID (for same CASEID and FDA_DT).The “primary suspected (PS)” restriction was consistently applied post-deduplication, retaining only data with PS in the DRUG table’s ‘Role_Code’, excluding non-PS records (documented in the analytical codebook).Role codes (DRUG table) were handled per FDA guidelines: only PS (Role_Code = PS) was included (non-PS codes excluded). Post-deduplication, reports without PS were excluded; missing/ambiguous codes were treated as non-PS or resolved via FDA’s glossary (FDA, 2024), with rules consistently applied and documented.

Dose and onset-time information extracted from the FAERS database was systematically screened prior to subgroup analysis. Records with missing, inconsistent, or biologically implausible dose or onset−time data were excluded from the corresponding stratified analyses. Cases with ambiguous daily dose, unrealistic ultrahigh/low dose values, or undefined onset time intervals were categorized as non−evaluable and excluded from subsequent analysis. Following data cleaning, only valid evaluable cases after data cleaning were included in dose−stratified and onset−time subgroup analyses, and the corresponding number of evaluable participants was reported for each subgroup.

Data Analysis: Four unequal disproportionality analysis methods were employed to identify associations between drugs and ADRs: Reporting Odds Ratio (ROR), Proportional Reporting Ratio (PRR), Bayesian Confidence Propagation Neural Network (BCPNN), and Multinomial Gamma-Poisson Shrinker (MGPS). All disproportionality analyses were constructed based on a standard 2 × 2 contingency table as shown in **Table 1**. All spontaneous adverse event reports retrieved from the FAERS database were used as the background comparator for disproportionality estimation. The detailed formulas and positive signal threshold criteria for each disproportionality analysis method were clearly defined. Generally, a value exceeding the threshold indicates a potential positive safety signal, and a larger corresponding value suggests a stronger signal strength. The reporting odds of coagulation dysfunction events for each agent were calculated as the ratio of the number of case reports associated with coagulation dysfunction to the number of reports documenting all other adverse events for the same drug. Data management and statistical analysis were performed using Microsoft Excel 2021(Microsoft Corporation, Redmond, Washington, United States) and R software version 4.4.2. The R package “ggplot2” was used to generate forest plots of positive signals, combined bar charts of outcome indicators, and box plots of onset time. Considering the limited number of coagulation−dysfunction reports for omadacycline and eravacycline, subgroup analyses stratified by age, gender, dose, clinical outcome and onset time were only performed for exploratory and descriptive purposes. No definitive clinical or causal conclusions were drawn from these limited subgroup data.

**Table 1:** A two-by-two contingency table and detailed formulas for disproportionality analysis.

	Target adverse drug event	Other adverse drug events	Sums
Target drug	a	b	a+b
All other drugs	c	d	c+d
Sums	a+c	b+d	a+b+c+d

Algorithms	Equation		Criteria
ROR	$\text{ROR}=\frac{\text{a}/\text{b}}{\text{c}/\text{d}}$ $95\%\text{CI}={\text{e}}^{\ln\left(\text{ROR}\right)\pm 1.96\sqrt{\frac{1}{\text{a}}+\frac{1}{\text{b}}+\frac{1}{\text{c}}+\frac{1}{\text{d}}}}$		ROR 95%CI Lower limit > 1, N ≥ 2
PRR	$\text{PRR}=\frac{\text{a}/(\text{a}+\text{c})}{\text{b}/(\text{b}+\text{d})}$ $\chi^{2}=\frac{{\left(\text{ad}-\text{bc}\right)}^{2}(\text{a}+\text{b}+\text{c}+\text{d})}{\left(\text{a}+\text{b})(\text{c}+\text{d})(\text{a}+\text{c})(\text{b}+\text{d}\right)}$		PRR ≥ 2, χ² ≥ 4, N ≥ 3
BCPNN	$\text{IC}=\log_{2}\left(\frac{\text{a}(\text{a}+\text{b}+\text{c}+\text{d})}{\left(\text{a}+\text{c})(\text{a}+\text{b}\right)}\right)$ ${\text{IC}}_{025}={\text{e}}^{\ln\left(\text{IC}\right)-1.96\sqrt{\frac{1}{\text{a}}+\frac{1}{\text{b}}+\frac{1}{\text{c}}+\frac{1}{\text{d}}}}$		IC025 > 0
MGPS	$\text{EBGM}=\frac{\text{a}(\text{a}+\text{b}+\text{c}+\text{d})}{\left(\text{a}+\text{c})(\text{a}+\text{b}\right)}$ ${\text{EBGM}}_{05}={\text{e}}^{\ln\left(\text{EBGM}\right)-1.64\sqrt{\frac{1}{\text{a}}+\frac{1}{\text{b}}+\frac{1}{\text{c}}+\frac{1}{\text{d}}}}$		EBGM05 > 2, N > 0

The formulas to calculate the signal strength are as follows: a, number of reports containing both the target drug and the target adverse drug event; b, number of reports containing other adverse drug events of the target drug; c, number of reports containing the target adverse drug event of other drugs; d, number of reports containing other drugs and other adverse drug events. 95% CI, 95% confidence interval; N, the number of reports; χ2, chi-squared; ROR, reporting odds ratio; PRR, proportional reporting ratio; BCPNN, Bayesian confidence propagation neutral network; MGPS, multi-item gamma Poisson shrinker; IC, information component; IC025, the lower limit of the 95% CI of the IC; E (IC), the IC expectations; V (IC), the variance of IC; EBGM, empirical Bayesian geometric mean; EBGM05, the lower limit of the 95% CI of EBGM.

## 3 Results

### 3.1 Descriptive analysis of basic information in adverse event reports

**Figure 1:**
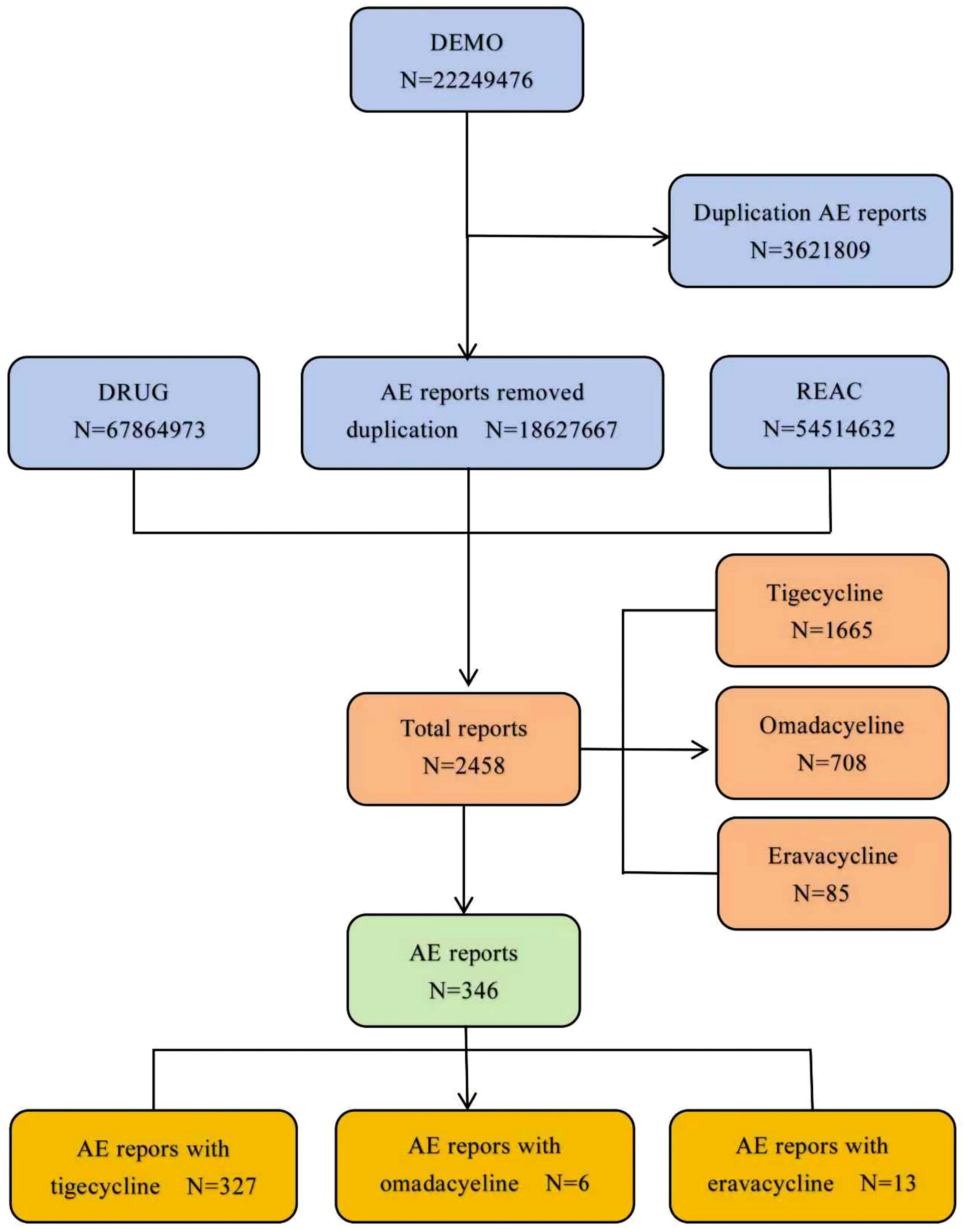
Flow chart for coagulation dysfunction events associated with novel tetracycline-class drugs from the FDA adverse event reporting system (FAERS).

**Figure 2:**
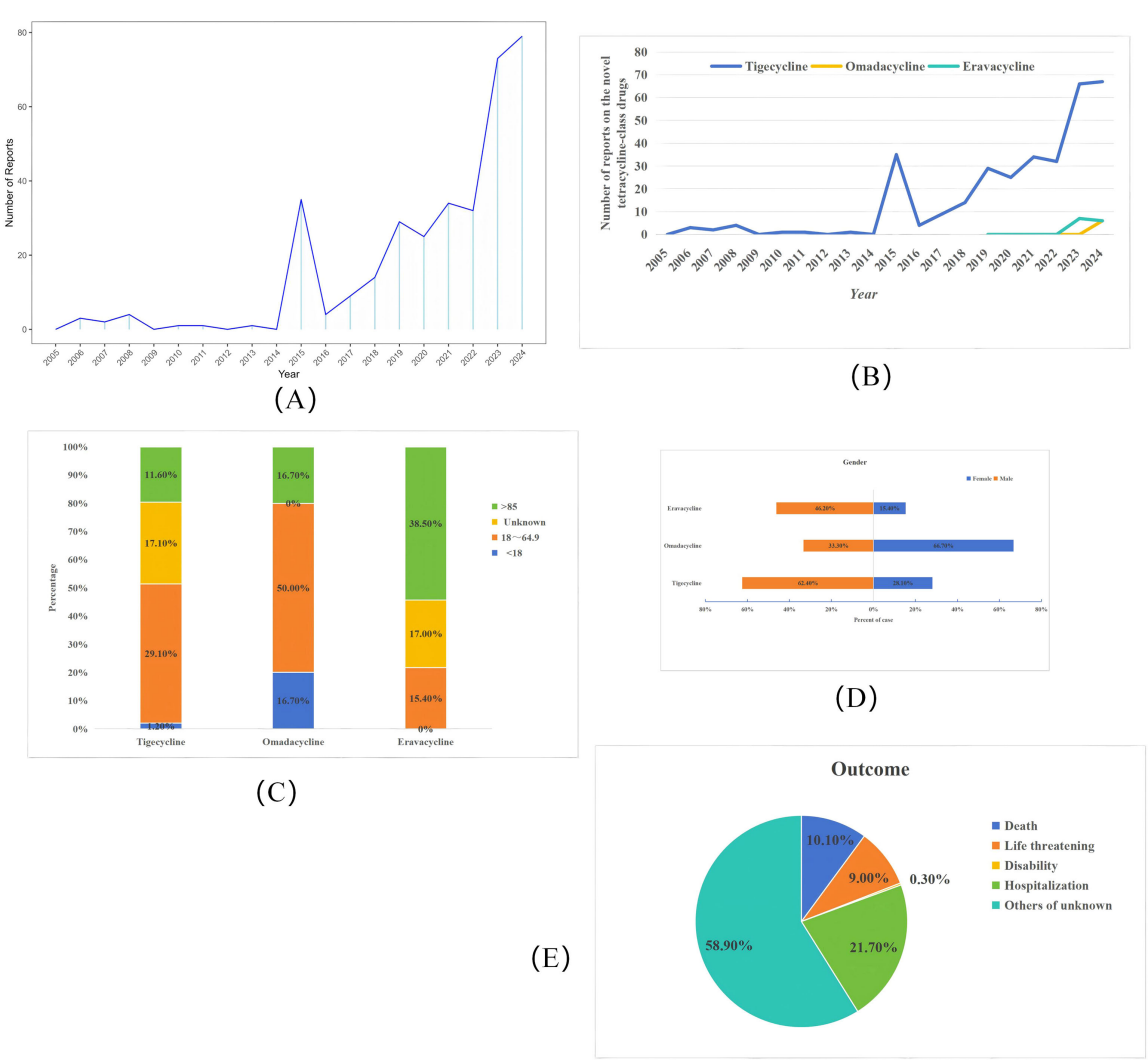
Annual increase in AEs associated with drug-induced coagulation dysfunction and clinical characteristics of the patients. **(A)** Overall growth trend; **(B)** growth trend of each individual drug; **(C)** age distribution of patients; **(D)** gender distribution of the patients; **(E)** patient outcomes;.

Between January 2004 to December 2024, a total of 22,249,476 adverse events associated with novel tetracyclines were retrieved from the FAERS database. After deduplication and rigorous screening, a total of 2,458 eligible adverse event were included in the final dataset, among which 1,665 were related to tigecycline, 708 to omadacycline, and 85 to eravacycline. Ultimately, 346 patients with coagulation abnormalities correlated with novel tetracyclines were enrolled in the present analysis (**Figure 1**). Among the included population, 327 cases were associated with tigecycline, 6 with omadacycline, and 13 with eravacycline. Baseline demographic and clinical profiles, including reporting regions, patient age, gender, and therapeutic indications, are summarized in **Table 2** and visualized in **Figure 2**.

**Table 2:** Clinical characteristics of adverse event reports for coagulation dysfunction associated with novel tetracycline-class drugs [case (%)].

Characteristics		Tigecycline	Omadacycline	Eravacycline
Gender	Female	92 (28.1%)	4 (66.7%)	2 (15.4%)
	Male	204 (62.4%)	2 (33.3%)	6 (46.2%)
	Unknown	31 (9.5%)	0 (0%)	5 (38.5%)
Age	<18	4(1.2%)	1 (16.7%)	0 (0%)
	18∼64.9	95(29.1%)	3 (50.0%)	2 (15.4%)
	65∼85	134 (41.0%)	1 (16.7%)	6 (46.2%)
	>85	38 (11.6%)	1 (16.7%)	0 (0%)
	Unknown	56 (17.1%)	0 (0%)	5 (38.5%)
Occupation of the reporters	health professional	94(28.7%)	2 (33.3%)	2(15.4%)
	Others	228(69.8%)	4 (66.7%)	11 (84.6%)
	Unknown	5 (1.5%)	0 (0%)	0 (0%)
Year	2004-2010	10(3.0%)	0 (0%)	0 (0%)
	2011-2015	37(11.3%)	0 (0%)	0 (0%)
	2016-2020	81(24.8%)	0 (0%)	0 (0%)
	2021-2024	199(60.9%)	6 (100%)	13(100%)
Adaptation disease(top 5)		Pneumonia 57 (17.4%)	Pneumonia 2 (33.3%)	Mycobacterium abscessus infection 6 (46.2%)
		Anti-infective therapy 51 (15.6%)	Bacteremia 1 (16.7%)	Anti-infective therapy 3 (23.1%)
		Infection 40 (12.2%)	Diabetic foot infection 1 (16.7%)	Empyema 1 (7.7%)
		Acinetobacter infection 16 (4.9%)	Mycobacterial infection 1 (16.7%)	Bacteremia 1 (7.7%)
		Abdominal infection 14 (4.3%)	Pneumonia Bacterial 1 (16.7%)	Klebsiella infection 1 (7.7%)
Outcome	Death	31 (9.5%)	1 (16.67%)	4 (30.8%)
	Life threatening	30 (9.2%)	0 (0%)	1 (7.7%)
	Disability	1 (0.3%)	0 (0%)	0 (0%)
	Hospitalization	70 (21.4%)	3 (50%)	2 (15.4%)
	Others	170(52.0%)	0 (0%)	5 (38.5%)
	Unknown	25 (7.6%)	2 (33.33%)	1 (7.7%)
Reporting region	Africa	1 (0.3%)	0 (0%)	0 (0%)
	Europe	80(27.4%)	0 (0%)	2 (15.4%)
	Asian	217(66.3%)	4 (66.7%)	5(38.5%)
	Oceania	0 (0%)	0 (0%)	0 (0%)
	America	0 (0.0%)	2(33.3%)	6 (46.2%)
	Unknown	0 (0%)	0 (0%)	0 (0%)

### 3.2 Disproportionality analysis of coagulation-related adverse events associated with novel tetracycline-class drugs

#### 3.2.1 Disproportionate reporting signal for coagulation disorders associated with novel tetracycline-class drugs

Quantitative disproportionality analysis identified pharmacovigilance signals for coagulation abnormalities in both tigecycline and eravacycline with ROR values of 46.85 (95%CI: 42.29–51.91) and 37.16 (95%CI: 22.28–61.97), respectively. In contrast, omadacycline did not yield any statistically significant disproportionate signals pertaining to coagulation dysfunction. Results are shown in **Table 3**.

**Table 3:** The signal intensity of coagulation dysfunction associated with novel tetracycline-class drugs.

Drug	Report number	ROR (95% CI)	PRR (95% CI)	MGPS (EBGM05)	BCPNN (IC025)
Tigecycline	327	46.85 (42.29 - 51.91)^*^	42.2 (42.11-42.19)^*^	38.62 ^*^	5.24 ^*^
Omadacycline	6	1.58 (0.79 - 3.17)	1.58 (0.89-2.27)	0.88	-0.3
Eravacycline	13	37.16 (22.28 - 61.97)^*^	34.17 (33.71-34.64)^*^	22.27 ^*^	4.36 ^*^

* indicates a positive signal.

**Figure 3:**
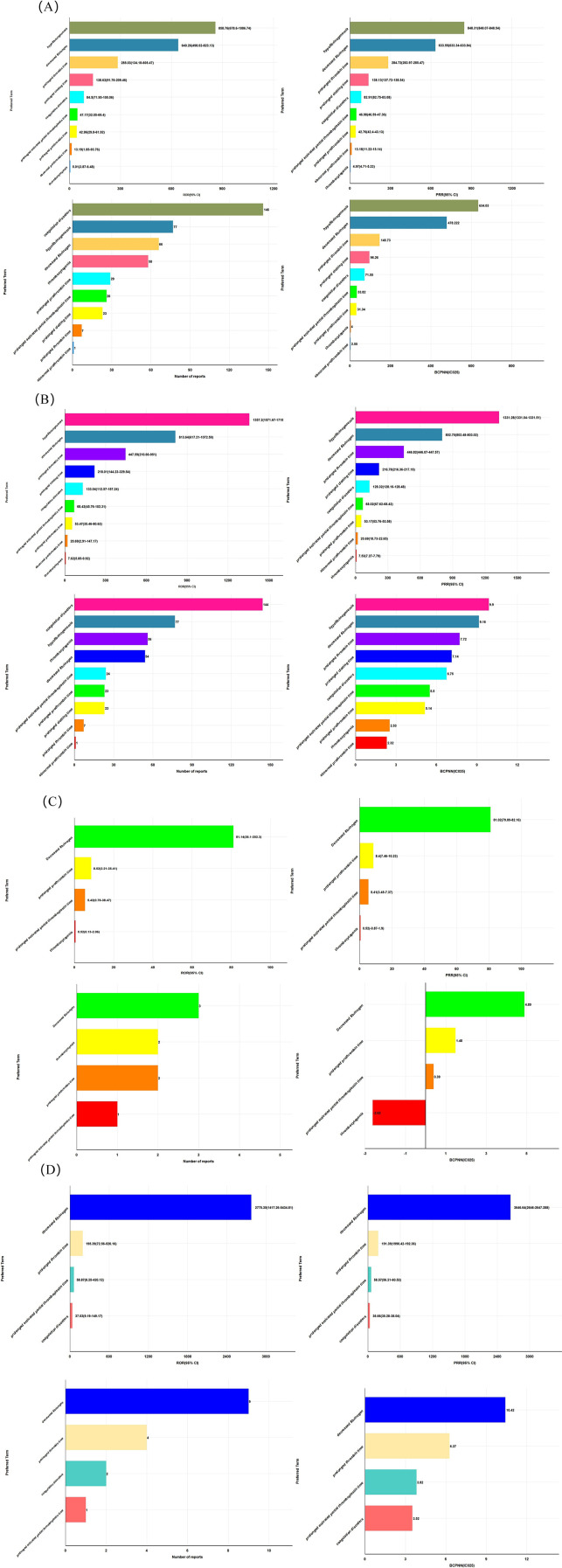
**(A)** Positive signals associated with the novel tetracycline-class drugs; **(B)** Positive signals associated with tigecycline; **(C)** positive signals associated with omacycline; **(D)** positive signals associated with eravacycline.

To further characterize the hematological profiles of coagulation-related adverse events, we summarized the most frequently reported coagulation abnormalities associated with novel tetracycline-class antibiotics. For tigecycline, the commonly reported coagulation abnormalities primarily encompassed hypofibrinogenemia, prolonged activated partial thromboplastin time (APTT), prolonged thrombin time (TT), prolonged prothrombin time, abnormal prothrombin time, prolonged clotting time, coagulation disorders, decreased fibrinogen, and thrombocytopenia. For omadacycline, relevant coagulation manifestations mainly included hypofibrinogenemia, prolonged activated partial thromboplastin time (APTT), and prolonged prothrombin time. In terms of eravacycline, frequently documented adverse events involved reduced fibrinogen concentration, prolonged activated partial thromboplastin time (APTT),prolonged prothrombin time, and coagulation disorders. As shown in **Figure 3**, hypofibrinogenemia and prolonged PT were the predominant coagulation abnormalities across the three novel tetracycline agents. (**Figure 3**; **Table 4**).

**Table 4:** The signal intensity of adverse events related to novel tetracycline-class drugs coagulation dysfunction.

Drug name	Preferred term (PT)	Report number	ROR (95% CI)	PRR (95% CI)	BCPNN (IC025)	MGPS (EBGM05)
Tigecycline	Hypofibrinogenemia	77	1357.2 (1071.67-1718.81)^*^	1331.28 (1331.04-1331.51)^*^	9.9^*^	994.32^*^
	Prolonged activated partial thromboplastin time	24	68.42 (45.76 - 102.31)^*^	68.02 (67.62-68.42)^*^	5.5^*^	48.34^*^
	Prolonged thrombin time	7	447.59 (210.66 - 951)^*^	446.82 (446.07-447.57)^*^	7.72^*^	230.21^*^
	Prolonged prothrombin time	23	53.47 (35.46 - 80.62)^*^	53.17 (52.76-53.58)^*^	5.14^*^	37.56^*^
	Abnormal prothrombin time	1	20.69 (2.91-147.17)^*^	20.69 (18.73-22.65)^*^	2.32^*^	4^*^
	Prolonged clotting time	23	218.01 (144.23-329.54)^*^	216.78 (216.36-217.19)^*^	7.14^*^	151^*^
	Coagulation disorders	144	133.04 (112.57 - 157.24)^*^	128.32 (128.16-128.48)^*^	6.75^*^	110.53^*^
	Decreased fibrinogen	54	813.64 (617.21-1072.59)^*^	802.75 (802.48-803.02)^*^	9.16	601.26^*^
	Thrombocytopenia	56	7.62 (5.85- 9.92)^*^	7.53 (7.27-7.79)^*^	2.53^*^	6.04^*^
Omadacycline	Thrombocytopenia	2	0.52 (0.13- 2.06)^*^	0.52 (-0.87-1.90)^*^	-2.62	0.16
	Prolonged activated partial thromboplastin time	1	5.42 (0.76-38.47)^*^	5.41 (3.45-7.37)^*^	0.39^*^	1.05
	Prolonged prothrombin time	2	8.85 (2.21-35.41)^*^	8.84 (7.46-10.23)^*^	1.48^*^	2.77^*^
	Decreased fibrinogen	3	81.14 (26.1-252.23)^*^	81.02 (79.89-82.16)^*^	4.89^*^	31.27^*^
Eravacycline	Decreased fibrinogen	9	2775.35 (1417.26 - 5434.81)^*^	2646.64 (2646-2647.28)^*^	10.42^*^	1494.14^*^
	Prolonged activated partial thromboplastin time	1	58.87 (8.25-420.12)^*^	58.57 (56.21-60.52)^*^	3.82^*^	11.31^*^
	Prolonged thrombin time	4	195.39 (72.56-526.16)^*^	191.39 (190.42-192.36)^*^	6.27^*^	83.49^*^
	Coagulation disorders	2	37.03 (9.19-149.17)^*^	36.66 (35.28-38.04)^*^	3.52^*^	11.43^*^

* indicates a positive signal.

#### 3.2.2 Subgroup disproportionality analysis of adverse coagulation events associated with novel tetracycline-class drugs

##### 3.2.2.1 Dosage subgroup disproportionality analysis

**Figure 4:**
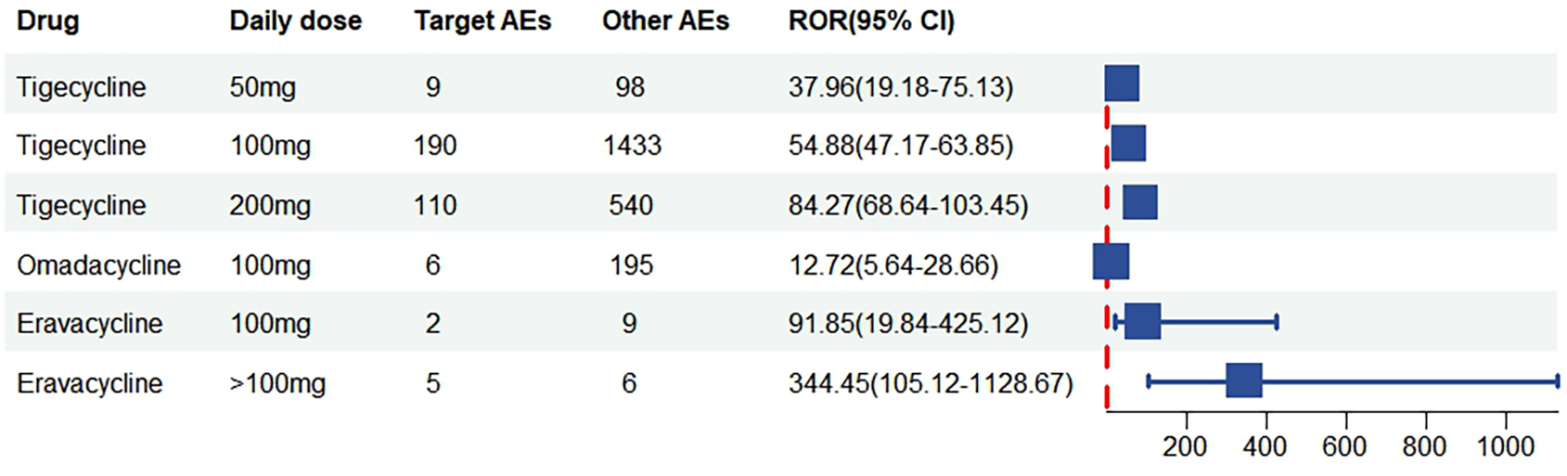
The signal intensity of adverse events related to coagulation disorders associated with novel tetracycline-class drugs in terms of daily dose.

The daily dose was calculated by multiplying the dose and dosing frequency provided in the database, and signal values were analyzed within subgroups based on the daily dose. See **Table 5**; **Figure 4**.

**Table 5:** The signal intensity of adverse events related to coagulation disorders associated with novel tetracycline-class drugs in terms of daily dose.

Drug	Daily dose	Evaluable case number	Target AEs	Other AEs	ROR (95% CI)	PRR (95% CI)	MGPS (EBGM05)	BCPNN (IC025)
Tigecycline	50mg	39	9	98	37.96 (19.18 - 75.13)^*^	34.85 (34.23-39.48)^*^	19.68 ^*^	4.17 ^*^
	100mg	635	190	1433	54.88 (47.17 - 63.85)^*^	48.57 (48.44-48.71)^*^	42.73 ^*^	5.38 ^*^
	200mg	259	110	540	84.27 (68.64 - 103.45)^*^	70.17 (70-70.34)^*^	59.06 ^*^	5.84 ^*^
Omadacycline	100mg	77	6	195	12.72 (5.64 - 28.66)^*^	12.37 (11.58-13.86)^*^	6.27 ^*^	2.52 ^*^
	150mg	18	0	60	NA	NA	NA	NA
	200mg	34	0	84	NA	NA	NA	NA
Eravacycline	100mg	4	2	9	91.85 (19.84 - 425.12)	75.33 (74.08-76.59)	20.9 ^*^	4.37^*^
	>100mg	5	5	6	344.45 (105.12 - 1128.67)^*^	188.34 (187.69-188.98)^*^	69.76 ^*^	6.11^*^

* indicates a positive signal.

##### 3.2.2.2 Subgroup disproportionality analysis of outcome measures

**Figure 5:**
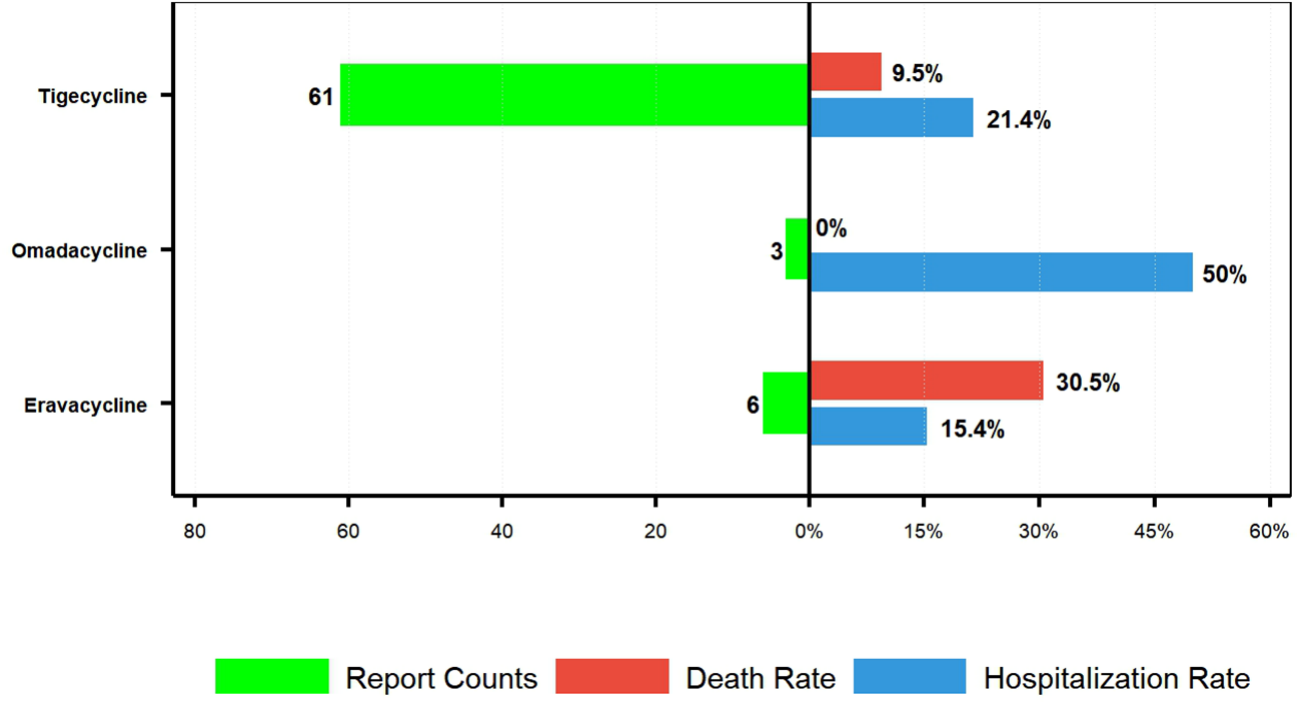
Proportion of coagulation dysfunction reports associated with novel tetracycline-class drugs stratified by clinical outcome.

By statistically analyzing the mortality and life-threatening outcomes among the outcome measures provided in the database, the subgroup signal values were assessed based on the statistical results. The findings are presented in **Tables 6**, **7**; **Figure 5**.

**Table 6:** Disproportionate reporting signal intensity of coagulation dysfunction associated with novel tetracycline-class drugs across clinical outcome indicators.

Drug	Target AEs	Other AEs	ROR (95% CI)	PRR (95% CI)	MGPS (EBGM05)	BCPNN (IC025)
Tigecycline	74	1049	29.17 (23.04 - 36.93)^*^	27.32 (27.1-27.54)^*^	22.41^*^	4.43 ^*^
Omadacycline	0	131	NA	NA	NA	NA
Eravacycline	5	25	82.67 (31.65 - 215.95)^*^	69.06 (68.06-69.26)^*^	30.92^*^	4.82^*^

* indicates a positive signal.

**Table 7:** Proportion of coagulation dysfunction reports associated with novel tetracycline-class drugs stratified by clinical outcome indicators.

Drug	Number	Death (%)	Hospitalization (%)
Tigecycline	61	9.5	21.4
Omadacycline	3	0	50
Eravacycline	6	30.5	15.4

##### 3.2.2.3 Age subgroup disproportionality analysis

**Figure 6:**
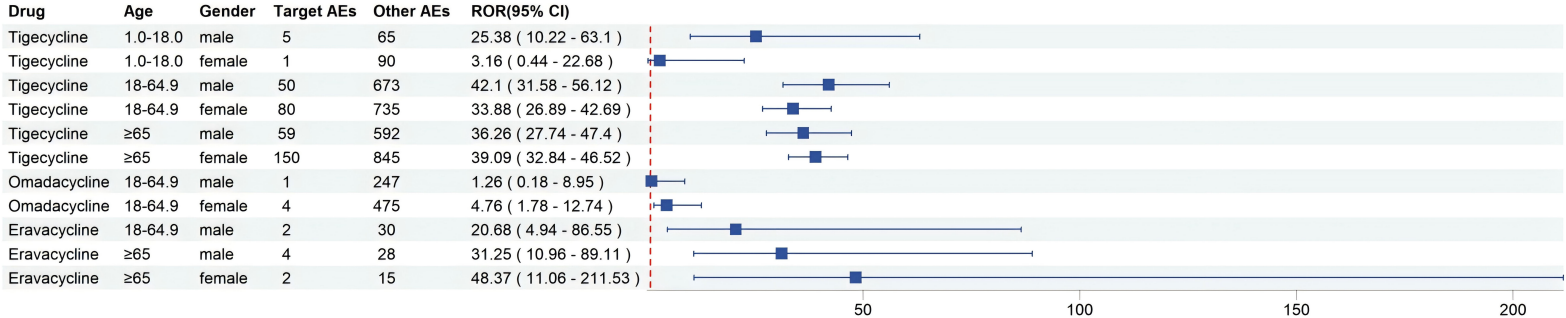
Disproportionate reporting signal of coagulation dysfunction associated with novel tetracycline-class drugs stratified by age and gender.

By statistically analyzing the age at onset and gender in the database, the subgroup signal values were evaluated based on the statistical results. The findings are presented in **Table 8**; **Figure 6**.

**Table 8:** Disproportionate reporting signal intensity of coagulation dysfunction associated with novel tetracycline-class drugs stratified by age and gender.

Drug	Age	Gender	Target AEs	Other AEs	ROR (95% CI)	PRR (95% CI)	MGPS (EBGM05)	BCPNN (IC025)
Tigecycline	1-18	Male	5	65	25.38 (10.22 - 63.1)^*^	23.65 (22.8-24.49)^*^	11.02^*^	3.33 ^*^
		Female	1	90	3.16 (0.44 - 22.68)^*^	3.14 (1.19-5.08)^*^	0.6	-0.41
	18-64.9	Male	50	673	42.1 (31.58 - 56.12)^*^	39.26 (38.99-39.52)^*^	30.8 ^*^	4.87 ^*^
		Female	80	735	33.88 (26.89 - 42.69)^*^	30.65 (30.45-30.86)^*^	25.18 ^*^	4.6^*^
	≥65	Male	59	592	36.26 (27.74 - 47.4)^*^	33.07 (32.82-33.31)^*^	26.34 ^*^	4.65^*^
		Female	150	845	39.09 (32.84 - 46.52)^*^	33.34 (33.2-33.49)^*^	28.63 ^*^	4.8 ^*^
Omadacycline	1-18	Male	NA	NA	NA	NA	NA	NA
		Female	NA	NA	NA	NA	NA	NA
	18-64.9	Male	1	247	1.26 (0.18 - 8.95)	1.25 (-0.7-3.21)	0.24	-1.72
		Female	4	475	4.76 (1.78 - 12.74)^*^	4.73 (3.76-5.71)^*^	2.08 ^*^	0.94^*^
	≥65	Male	NA	NA	NA	NA	NA	NA
		Female	NA	NA	NA	NA	NA	NA
Eravacycline	1-18	Male	NA	NA	NA	NA	NA	NA
		Female	NA	NA	NA	NA	NA	NA
	18-64.9	Male	2	30	20.68 (4.94 - 86.55)^*^	19.45 (18.11-20.79)^*^	5.87 ^*^	2.54^*^
		Female	NA	NA	NA	NA	NA	NA
	≥65	Male	4	28	31.25 (10.96 - 89.11)^*^	27.47 (26.55-28.39)^*^	11.43^*^	3.39^*^
		Female	2	15	48.37 (11.06 - 211.53)^*^	42.8 (41.5-44.1)^*^	12.45^*^	3.62^*^

*indicates a positive signal.

### 3.3 Onset time of the tetracycline-related coagulation disorder adverse events

**Figure 7:**
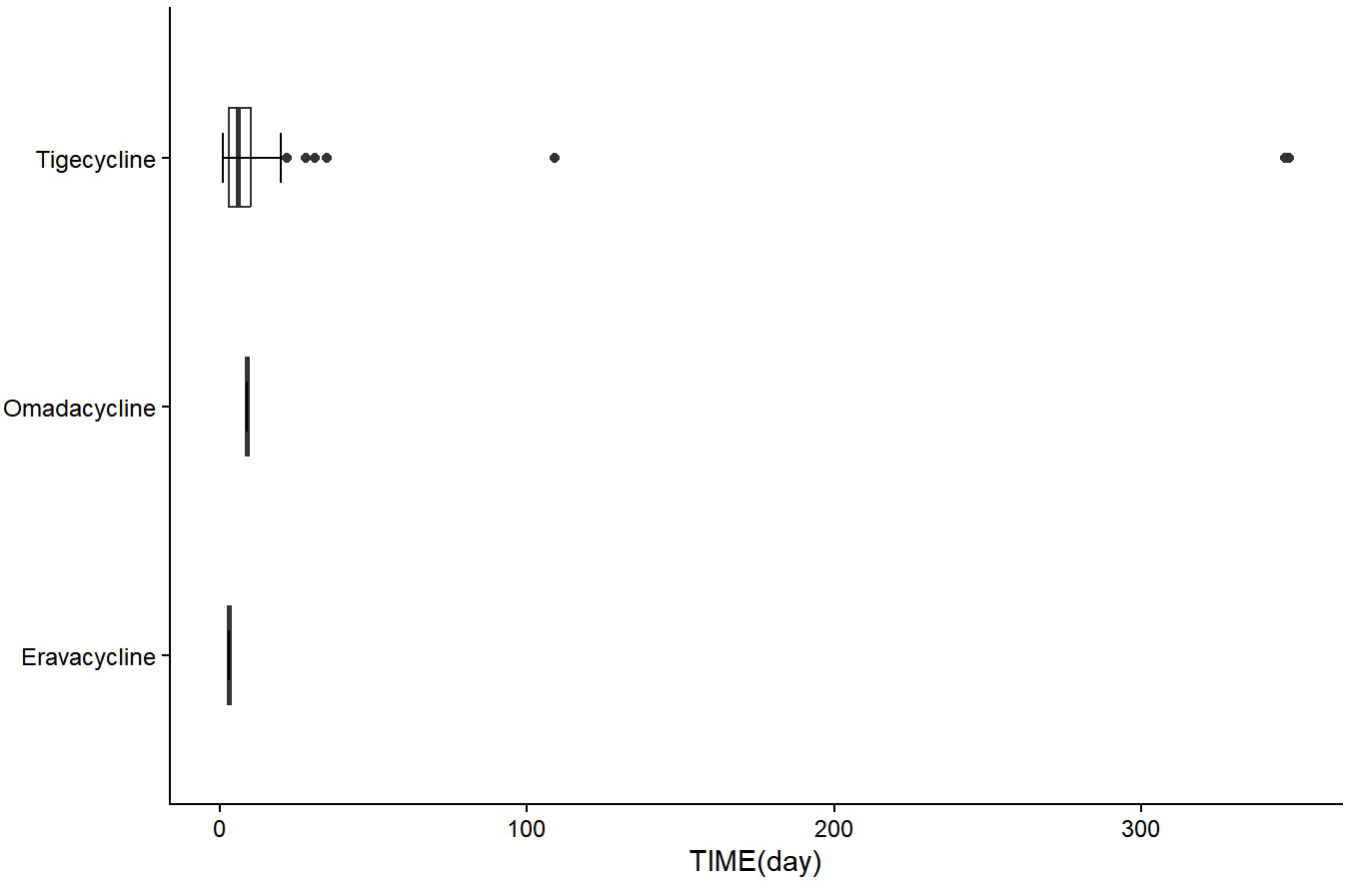
Onset time distribution of coagulation adverse events associated with novel tetracyclines. Outliers with an onset time beyond 300 days were observed, which were sporadic late-reported cases and did not affect the overall median onset trend.

Onset time of coagulation disorder adverse events was statistical analyzed based on evaluable cases, comprising 137 tigecycline-related cases, 6 omadacycline-related cases, and 2 eravacycline-related cases. In the evaluable cohort, tigecycline-related coagulation disorder events mainly occurred within 0–10 days, with a median onset time of 6 (1–109) days. The median onset time was 9 days for omadacycline and 3 days for eravacycline, respectively (**Figure 7**).

## 4 Discussion

### 4.1 Mechanisms of the novel tetracycline-induced coagulation disorders

In recent years, novel tetracycline-class agents have occupied an important role in the management of multidrug-resistant bacterial infections, and their safety profiles have attracted increasing clinical attention, particularly concerning hematologic adverse reactions (Zhang et al., 2024). Notably, the underlying mechanisms linking novel tetracyclines to coagulation dysfunction have not been fully elucidated. Given the inherent limitations of FAERS spontaneous reporting data, the following mechanistic discussions are presented merely as exploratory and hypothesis-generating interpretations, rather than confirmed causal relationships.

Several biologically plausible mechanisms supported by existing literature may partly explain the potential association between novel tetracyclines and coagulation abnormalities. Primary evidence indicates that tigecycline can modulate leukocyte IL-6 expression; as IL-6 is a key regulator of fibrinogen synthesis, altered inflammatory cytokine levels may indirectly affect circulating fibrinogen concentrations (Fuller, 1985; Vasse et al., 1996). Another well-recognized pathway relates to intestinal microbiota disturbance: tigecycline may alter gut microbial composition, impair endogenous vitamin K synthesis, and subsequently compromise the activity of vitamin K-dependent coagulation factors (Lei et al., 2023). Additional speculation focuses on the structural characteristics of novel tetracyclines. Similar to traditional tetracyclines, tigecycline may interfere with hepatic ribosomal function, potentially affecting the synthesis of fibrinogen and other coagulation-related factors. Other proposed pathways, including autophagy regulation, immune complex-mediated fibrinogen alteration, and genetic susceptibility, remain insufficiently validated by direct clinical or experimental evidence and are therefore not elaborated further in this study (Fish and Neerman-arbez, 2012; Wang et al., 2014; Chen and Shi, 2018; Ma et al., 2018).

FAERS-based disproportionality analysis detected disproportional reporting signals of coagulation dysfunction for tigecycline and eravacycline. Clinical observations have noted sustained hypofibrinogenemia following a switch from tigecycline to eravacycline, while coagulation parameters appear to improve after conversion to omadacycline (Rausch et al., 2022). As a preliminary exploratory hypothesis, structural differences in the C7 fluorine substituent among novel tetracyclines may partially account for such inter-agent discrepancies. The fluorinated moiety of eravacycline may theoretically interfere with hepatic metabolic pathways related to perfluorinated compounds, which is hypothetically linked to altered fibrinogen biosynthesis (Wang and Zhang, 2022).

Overall, the mechanistic basis underlying the potential association between novel tetracyclines and coagulation dysfunction remains incompletely clarified. Considering the relatively limited post-marketing real-world data of omadacycline, longer-term pharmacovigilance and clinical observational studies are still needed to confirm its relevance to coagulation function. All mechanistic interpretations in the current analysis are exploratory and hypothesis-generating only, and cannot be regarded as definitive causal conclusions.

### 4.2 Reporting proportion of coagulopathy associated with novel tetracycline-class drugs

In this study, both tigecycline and eravacycline were found to be statistically significant associated with coagulation dysfunction, while no relevant disproportionate signals related to coagulation dysfunction were detected for omadacycline. These findings are consistent with those reported in previously published literatures (Rausch et al., 2022; Liang et al., 2024; Liu and Bai, 2024). Eravacycline carries a fluorine substitution at the C7 position and undergoes predominant hepatic metabolism; existing evidence suggests that these structural and metabolic feature may be hypothetically correlated with reduced fibrinogen synthesis (Wang and Zhang, 2022). In comparison, approximately 81.1% of omadacycline is excreted unchanged in feces, resulting in minimal hepatic exposure and presumably a weaker influence on fibrinogen production (Rausch et al., 2022). A multicenter study on concerning treatment regimens for Mycobacterium abscessus reported no episodes of hypofibrinogenemia or bleeding complications during omadacycline therapy (Morrisette et al., 2021).

Subgroup analysis of different coagulation-related events in this study revealed positive disproportionate signals of omadacycline for hypofibrinogenemia, prolonged activated partial thromboplastin time (APTT), and prolonged prothrombin time (PT). A systematic literature search of CNKI and PubMed databases was conducted using the keywords “omadacycline”, “hypofibrinogenemia”, “prolonged APTT” and “prolonged prothrombin time”, however, retrieved no relevant spontaneous adverse drug reaction reports were identified. This discrepancy may imply a potential false-positive signal from the FAERS database, or it may reflect clinically rare, underrecognized coagulation events that have not yet been widely documented (Pan, 2019). Notably, such coagulation-related events are not listed in the official drug labeling. These unreported signals may be hypothetically linked to hypersensitivity responses, suggesting that omadacycline could also correlate with altered coagulation function. This potential safety signal merits close clinical attention and requires further real-world verification.

### 4.3 Baseline data analysis

Baseline descriptive analyses revealed that adverse coagulation events associated with novel tetracyclines were predominantly submitted between 2021 and 2024. This temporal distribution may correspond to the marketing timeline of these agents and growing clinical awareness of their hematological adverse reactions. Geographically, most eligible reports originated from Asia, and non-medical personnel accounted for the largest proportion of submitters. Notably, the FAERS database only contains country-level reporting information, without detailed data regarding patient race or genetic background. Due to limitations related to reporting country and reporter heterogeneity, further research is needed to explore potential racial disparities in coagulation dysfunction associated with novel tetracycline-class antibiotics.

### 4.4 Subgroup disproportionality analysis

Children under the age of 18 are not advised to use tigecycline. However, children over the age of 8 can use it for infections for which there are no other medications (Bodmannk et al., 2012).It is unclear if omadacycline and eravacycline are safe and effective for children under the age of 18. However, neither is currently advised for children under the age of 8 due to their effects on tooth development. The safety of tigecycline in youngsters under the age of eighteen was actively monitored in the real world. The findings indicated that hypofibrinogenemia and impaired coagulation function were the more common adverse drug reactions (ADRs) (Li et al., 2025), consistent with the safety study results in people aged 18 and above (Shi et al., 2025). Subgroup disproportionality analysis by age, gender, and dosage revealed a higher proportion of male patients receiving tigecycline and eravacycline. The findings for tigecycline align with previous reports (Qin et al., 2022; Li et al., 2025). Coagulation disorders associated with tigecycline were most prevalent in the 65–85 age group, with males aged 18-64.9 years exhibiting proportion of reports than females in the same age range, consistent with the study by Liu et al. (2021). Tigecycline is rarely utilized in clinical practice for individuals under the age of eighteen, yet reporting signal are nevertheless present (Gui et al., 2019; Li et al., 2025). Nevertheless, two other studies similarly indicated that age was not correlated with coagulation dysfunction associated with tigecycline (Treml et al., 2021; Xv and Tan, 2024).In line with those previous observations, the present descriptive exploratory analysis detected coagulation-related disproportionality signals across all age groups for tigecycline. Further research is required to explore whether age is correlated with reduced fibrinogen levels. Two double-blind, placebo-controlled phase I clinical trials looked at how age and gender affected the pharmacokinetics of omadacycline administered orally and intravenously to healthy volunteers (Villano et al., 2016). The findings shows that despite the higher amounts of omadacycline exposure among female participants in both trials, the impact on clinical applications was minimal. Therefore, it is not possible to adjust the dosage of omadacycline based on the patient’s age or gender. Additionally, this study discovered that females between the ages of 18-64.9 had disproportionate reporting signal of omadacycline, consistent with Amer El Ghali’s study (El Ghali et al., 2023), which included a higher proportion of females. Tigecycline and eravacycline exhibited stronger disproportionate reporting signals in male patients over 65 years old. This pattern may be partially explained by the frequent clinical use of tigecycline and eravacycline for severe infections associated with multidrug-resistant bacteria, clinical scenarios that are more prevalent among elderly individuals. The percentage of female patients on omadacycline and tigecycline was comparatively high in this study, consistent with VILLANO S’s study (Villano et al., 2016), inconsistencies with Qin’s study (Qin et al., 2022).This finding suggests that gender exerts no meaningful influence on the risk of novel tetracycline−associated coagulation dysfunction. It’s interesting to note that a comparatively high percentage of male patients are on eravacycline, which has not been documented in earlier research. However, this observation is based on a very small number of cases and thus should not be overinterpreted. Tigecycline and eravacycline showed a disproportionate reporting pattern of coagulation-related adverse events at higher daily doses. Literature studies indicate that coagulation abnormalities and hypofibrinogenemia induced by high-dose maintenance therapy exceed those observed at recommended doses (Campany-herrero et al., 2020; Hu et al., 2020).Omadacycline, however, showed disproportionate reporting signal at a daily dose of 100 mg, a finding not previously documented in prior research. In the clinical outcomes subgroup analysis, the risk-of-recurrence (ROR) signal for mortality and life-threatening events in tigecycline users was [29.17 (23.04 - 36.93)],No deaths or life-threatening outcomes related to coagulation disorders were detected with Omadacycline. This observation may be explained by the fact that severe polymicrobial infections, which represent the primary clinical indications for tigecycline and eravacycline and are reportingly associated with coagulation abnormalities, inherently present elevated baseline mortality. Due to the extremely small case counts for omadacycline (n=6) and eravacycline (n=13), all subgroup analyses for these two agents, including age, gender, dose, and clinical outcome strata, are limited by poor statistical stability. Estimates of ROR, PRR, and Bayesian shrinkage metrics may be highly unstable under sparse data, and observed subgroup patterns are likely driven by a very small number of individual cases. Therefore, all subgroup findings for omadacycline and eravacycline are regarded as exploratory rather than confirmatory, and no robust clinical or mechanistic conclusions can be drawn from these limited data.

### 4.5 Disproportionality analysis of coagulation adverse event onset time

Onset pattern analysis suggested that tigecycline-related adverse events tended to cluster within 0–10 days after administration, which was broadly comparable with observations from previous literature (Fang et al., 2020). The median onset of tigecycline-associated coagulation events was approximately 6 days; notably, several late-onset outliers with an onset interval exceeding 300 days were also identified in the distribution (**Figure 7**). Such extreme delayed events are likely attributable to inherent limitations of spontaneous reporting databases, including delayed case reporting, prolonged hospitalization, complex underlying comorbidities, and concomitant polypharmacy. These sporadic outliers do not reflect the typical temporal trend and were not used to interpret the overall onset pattern.

For eravacycline, the onset distribution appeared to peak at day 3, which seemed earlier than the median onset of 10 days reported by Rausch et al (Morrisette et al., 2021).This observed discrepancy may partly relate to the relatively broader inclusion criteria in our study, which included patients from multiple medical centers and with diverse infectious pathogens, while the referenced study targeted a more specific infection population. Regarding omadacycline, peak onset was noted around day 9; this exploratory observation has rarely been documented in existing literature. Notably, the analyses for omadacycline (n=6) and eravacycline (n=13) were merely descriptive and exploratory given the limited case numbers. Due to the small sample size, the observed peak onset time as well as age- and gender-related distribution patterns should be interpreted with great caution. These findings are not robust or generalizable, and should only be considered hypothesis-generating observations rather than definitive clinical patterns.

### 4.6 Limitation

Despite the adoption of comprehensive statistical analyses, several limitations of this study should be acknowledged. First, the FAERS database is inherently susceptible to reporting bias and incomplete data, and relevant confounding factors cannot be fully adjusted. Second, the potential interference of concomitant medications was not statistically assessed in this study. Third, thus, their confounding effects cannot be excluded. Third, owing to the relatively short marketing duration of omadacycline and eravacycline, their available post-marketing data remain limited compared with tigecycline, which may introduce statistical bias. Fourth, underlying patient comorbidities were not incorporated into the analysis due to database data deficiency, and such disease-related parameters may serve as potential confounding factors for coagulation abnormalities. Fifth, the lack of detailed concomitant medication information in the FAERS database. Coagulation-related adverse events are highly susceptible to interference from concurrent medications such as warfarin, heparin, and NSAIDs, which serve as major confounding variables. Without individual-level data on combined drug use, we could not perform stratification or multivariate adjustment to exclude the confounding effects of these anticoagulant/antiplatelet drugs. This unmeasured confounding may bias the disproportionality estimates and reduce the reliability of the ROR and PRR values for coagulation events associated with novel tetracyclines. Therefore, the detected signals should be regarded as hypothesis-generating rather than confirmatory evidence of causality, and our findings need further validation in controlled clinical studies with complete concomitant medication records. Collectively, these inherent limitations emphasize that the observed associations should be interpreted cautiously, and further prospective clinical studies are required to validate our findings.

## 5 Conclusions

This study performed a comprehensive analysis of coagulation-related adverse events linked to novel tetracycline-class agents. Disproportionality analyses identified robust reporting signals of coagulation dysfunction for tigecycline and eravacycline, whereas no general positive signal was detected for omadacycline, which aligns with the outcomes of previous studies. Nevertheless, subgroup analyses revealed discrete positive signals of hypofibrinogenemia, prolonged activated partial thromboplastin time, and prolonged prothrombin time associated with omadacycline. This subtle and previously unrecognized phenomenon merits clinical attention and requires further verification. Methodologically, the present study innovatively explored the clinical characteristics and disproportional reporting patterns of tetracycline-associated coagulation abnormalities through stratified subgroup analysis, an approach that remains underexplored in prior real-world studies. Collectively, these findings may offer evidence-based references for clinicians and pharmacovigilance policymakers to refine adverse event monitoring and facilitate the safe and rational clinical administration of novel tetracycline-class antibiotics.

## Data Availability

The original contributions presented in the study are included in the article/**Supplementary Material**. Further inquiries can be directed to the corresponding author/s.
