# Supplementary material for: Coagulation dysfunction events associated with novel tetracycline-class drugs: a disproportionality and Bayesian analysis based on the FAERS database
Source: Front Antibiot. 2026 Jun 5;5:1781902. doi: 10.3389/frabi.2026.1781902 (PMC13279702; doi:10.3389/frabi.2026.1781902)
Supplement: Supplementary file 1 [file Table1.docx]

TABLE 1 Raw data of signal intensity of coagulation dysfunction induced by novel tetracycline-class drugs

| Drug | a | b | c | d |
| --- | --- | --- | --- | --- |
| Tigecycline | 409 | 3619 | 130539 | 54120034 |
| Omadacycline | 8 | 2091 | 130940 | 54121562 |
| Eravacycline | 16 | 178 | 130932 | 54123475 |

TABLE 2 Raw data of signal intensity of adverse events related to novel tetracycline-class drugs coagulation dysfunction

| Drug name | Preferred term (PT) | a | b | c | d |
| --- | --- | --- | --- | --- | --- |
| Tigecycline | Hypofibrinogenemia | 77 | 3951 | 779 | 54249794 |
|  | Prolonged activated partial thromboplastin time | 24 | 4004 | 4752 | 54245821 |
|  | Prolonged thrombin time | 7 | 4021 | 211 | 54250362 |
|  | Prolonged prothrombin time | 23 | 4005 | 5826 | 54244747 |
|  | Abnormal prothrombin time | 1 | 4027 | 651 | 54249922 |
|  | Prolonged clotting time | 23 | 4005 | 1429 | 54249144 |
|  | Coagulation disorders | 144 | 3884 | 15114 | 54235459 |
|  | Decreased fibrinogen | 54 | 3974 | 906 | 54249667 |
|  | Thrombocytopenia | 56 | 3972 | 100156 | 54150417 |
| Omadacycline | Thrombocytopenia | 2 | 2097 | 100210 | 54152292 |
|  | Prolonged activated partial thromboplastin time | 1 | 2098 | 4775 | 54247727 |
|  | Prolonged prothrombin time | 2 | 2097 | 5847 | 54246655 |
|  | Decreased fibrinogen | 3 | 2096 | 957 | 54251545 |
| Eravacycline | Decreased fibrinogen | 9 | 185 | 951 | 54253456 |
|  | Prolonged activated partial thromboplastin time | 1 | 193 | 4775 | 54249632 |
|  | Prolonged thrombin time | 4 | 190 | 5845 | 54248562 |
|  | Coagulation disorders | 2 | 192 | 15256 | 54239151 |

TABLE 3 Raw data of signal intensity of adverse events related to coagulation disorders associated with novel tetracycline-class drugs in terms of daily dose

| Drug | Daily dose | a | b | c | d |
| --- | --- | --- | --- | --- | --- |
| Tigecycline | 50mg | 9 | 98 | 130939 | 54123555 |
|  | 100mg | 190 | 1433 | 130758 | 54122220 |
|  | 200mg | 110 | 540 | 130838 | 54123113 |
| Omadacycline | 100mg | 6 | 195 | 130942 | 54123458 |
|  | 150mg | NA | NA | NA | NA |
|  | 200mg | NA | NA | NA | NA |
| Eravacycline | 100mg | 2 | 9 | 130946 | 54123644 |
|  | ＞100mg | 5 | 6 | 130943 | 54123647 |

TABLE 4 Raw data of signal intensity of related coagulation dysfunction caused by novel tetracycline-class drugs in terms of outcome indicators

| Drug | a | b | c | d |
| --- | --- | --- | --- | --- |
| Tigecycline | 74 | 1049 | 130874 | 54122604 |
| Omadacycline | NA | NA | NA | NA |
| Eravacycline | 5 | 25 | 130943 | 54123628 |

TABLE 5 Raw data of signal intensity of related coagulation dysfunction caused by the novel tetracycline-class drugs in terms of age and gender

| Drug | age | Gender | a | b | c | d |
| --- | --- | --- | --- | --- | --- | --- |
| Tigecycline | 1-18 | Male | 5 | 65 | 2919 | 963421 |
|  |  | Female | 1 | 90 | 3293 | 936264 |
|  | 18-64.9 | Male | 50 | 673 | 23675 | 13415094 |
|  |  | Female | 80 | 735 | 22503 | 7004849 |
|  | ≥65 | Male | 59 | 592 | 18817 | 6846481 |
|  |  | Female | 150 | 845 | 21937 | 4830267 |
| Omadacycline | 1-18 | Male | NA | NA | NA | NA |
|  |  | Female | NA | NA | NA | NA |
|  | 18-64.9 | Male | 1 | 247 | 22582 | 7005337 |
|  |  | Female | 4 | 475 | 23721 | 13415292 |
|  | ≥65 | Male | NA | NA | NA | NA |
|  |  | Female | NA | NA | NA | NA |
| Eravacycline | 1-18 | Male | NA | NA | NA | NA |
|  |  | Female | NA | NA | NA | NA |
|  | 18-64.9 | Male | 2 | 30 | 22581 | 7005554 |
|  |  | Female | NA | NA | NA | NA |
|  | ≥65 | Male | 4 | 28 | 22083 | 4831084 |
|  |  | Female | 2 | 15 | 18874 | 6847058 |
